# Enhancing the color gamut of waveguide displays for augmented reality head-mounted displays through spatially modulated diffraction grating

**DOI:** 10.1038/s41598-024-59231-z

**Published:** 2024-04-17

**Authors:** Jae-Sang Lee, Seong-Hyeon Cho, Woo June Choi, Young-Wan Choi

**Affiliations:** 1 Department of Intelligent Semiconductor Engineering, Chung-Ang University, Seoul, South Korea; 2 Department of Electrical and Electronics Engineering, Chung-Ang University, Seoul, South Korea

**Keywords:** Displays, Micro-optics

## Abstract

Augmented reality (AR) applications require displays with an extended color gamut to facilitate the presentation of increasingly immersive content. The waveguide (WG) display technology, which is typical AR demonstration method, is a critical constraint on the color gamut of AR systems because of the intrinsic properties of the holographic optical elements (HOEs) used in this technology. To overcome this limitation, we introduce a method of spatially modulated diffractive optics that can expand the color gamut of HOE-based WG displays. This approach involves spatial modulation using sub-pixelized HOEs, which enables the diffraction of red, green, and blue rays along identical directions. The proposed structure considers both the characteristics of the HOE and the wavelength sensitivity of the observer to optimize the color gamut. Consequently, an expanded color gamut was achieved. The results of the theoretical and experimental analyses substantiate the effectiveness and practicality of this method in enhancing the color gamut of HOE-based WG displays. Thus, the proposed method can facilitate the implementation of more immersive AR displays.

## Introduction

The rapid development of mobile devices, particularly smartphones, has coincided with the advancements in microprocessors, communication, and display technologies. Head-mounted displays (HMDs) with augmented reality (AR) features are gaining attention as next-generation mobile technologies^1–4^. Products such as the Apple Vision Pro have also garnered considerable interest, highlighting the pivotal role of optical see-through displays in popularizing AR-HMDs and distinguishing them from standard smartphones^5,6^.

Optical see-through displays require a transparent image combiner to effectively merge the real-world and virtual imagery. Image combiners are classified into two types: the birdbath type that employs macro-optical elements such as beam splitters, and the waveguide (WG) type, which is based on micro- or nano-optical elements such as diffraction gratings^7–9^. The WG-type image combiners present a more compact and lightweight design than the birdbath-type, enhancing user comfort and reducing the overall weight of the AR-HMDs^10–12^.

A WG-type display is composed of optical elements that utilize diffraction. Diffractive optical elements typically comprise a grating or a holographic structure. They are used as input or output couplers for the WG displays, functioning as couplers in and out of the WG display. This enables the virtual content from the projector to be delivered to the human eye through the WG^13,14^. Diffractive optical elements can be classified into surface, volume, and polarization volume type gratings. These elements have a wavelength-selective property, diffracting only light in a specific wavelength band for which they are designed while transmitting light at other wavelengths.

Surface type gratings, also known as surface relief gratings (SRGs), are fabricated using techniques such as e-beam patterning, etching, and nano-imprinting^15,16^. For example, Microsoft HoloLens 2 employs SRGs to deliver the virtual content to user eyes^13^. However, these SRGs face challenges such as susceptibility to dispersion effects, which can cause uneven color distribution and the appearance of a rainbow effect. Moreover, higher diffraction orders can lead to unwanted light leakage from the optical WG.

Volume-type gratings, known as volume-holographic gratings (VHGs), are characterized by a three-dimensional structure formed by periodic variations in the refractive index. The representative method for demonstrating VHGs is the use of photopolymer based holographic optical elements (HOEs)^10,17,18^. The coherent lights intersect on the HOE, creating a volumetric interference pattern, which forms a VHG by modulating the material’s refractive index. HOE can be implemented as reflective or transmissive elements. The ghost effect in VHGs is an inherent drawback of the transmission-type HOE, which is designed to diffract virtual content to the eye from the same side as the real world. This phenomenon is primarily caused by stray light, with dispersion also playing a role. However, stray light resulting from unintended diffraction of light from real objects is the principal factor contributing to ghost images. It is typically caused by unwanted diffraction orders or stray light interfering with the desired image^19^, and is not observed in reflective-type HOEs. Reflective volume holograms are typically fabricated by exposing photosensitive materials to laser light by using photopolymers as the medium. As compared with SRGs, HOEs present remarkable advantages such as reduced research and development costs and faster production, significantly promoting research efforts^13,17,19^.

The polarization volume grating (PVG) is a type of optical grating that selectively diffracts light according to its polarization state^20–23^.PVGs are liquid crystal based gratings that have a high diffraction efficiency close to 100%. They use the spatial variation of the optical anisotropy within a volume. This anisotropy allows the grating to interact differently with light of various polarization states, such as linear, circular, or elliptical polarization.

SRGs and VHGs employ the spatial period and slanted angle of the grating to determine the angle of light diffraction. However, these methods face limitations in full-color displays caused by the wavelength-dependent nature of diffraction. Hence, the color gamut, which represents the range of colors that a device or system can display, is restricted by the inherent wavelength selectivity in the WG displays that employ SRGs or VHGs. To overcome these challenges, SRGs have adopted compound microstructures to correct the chromatic aberrations^14^. However, ghost image issues are inevitable issue due to ambient light diffraction. Various methods have been proposed to address this issue, such as controlling the exposure time of the red (R), green (G), and blue (B) lasers or layered HOE^24–26^. Nonetheless, these approaches face several limitations. Unintended diffraction effects can occur when light of a different wavelength that was not recorded is incident on the HOEs. For example, if G light is incident on an HOE recorded with an R laser, it produces noise and distortion.

In this study, we introduce a novel approach that utilizes a spatially modulated diffraction grating (SMDG) with an HOE to enhance the color gamut. This method divides each pixel into multiple subpixels, which are each designed to record a specific wavelength. This is achieved by using a mask that blocks all the other regions while recording only a particular wavelength. Following fine-tuning using a nanoactuator, we obtained subpixel recordings across distinct areas. We then introduced three variants of SMDG HOEs: the first variant is a recorded HOE that considers R, G, and B individually by dividing the pixels into thirds (trisected HOE); the second variant is a trichromatic balanced-HOE (TCB-HOE) that accounts for the optical properties of the HOE; and the third variant is a tristimulus balanced-HOE (TSB-HOE) that takes into consideration both the HOE characteristics and the spectral response of a charge-coupled device (CCD) camera by functioning as a user in an experiment.

The remainder of this paper is organized as follows: Section "Methods" presents a detailed description of these principles and introduces three different types of HOE structures by using simulations and theoretical analyses. Section "Results" presents a comparative analysis of the diffraction and WG efficiencies of the three types of SMDG HOEs. Here, we present the intuitive AR implementation results demonstrating the enhancement of the color gamut and the quantitative analysis the color-filtered data of the three types of WG displays. Section "Discussion" discusses the obtained results, and Section "Conclusion" presents the conclusions.

## Methods

### Basic principle of SMDG HOE

**Figure 1:**
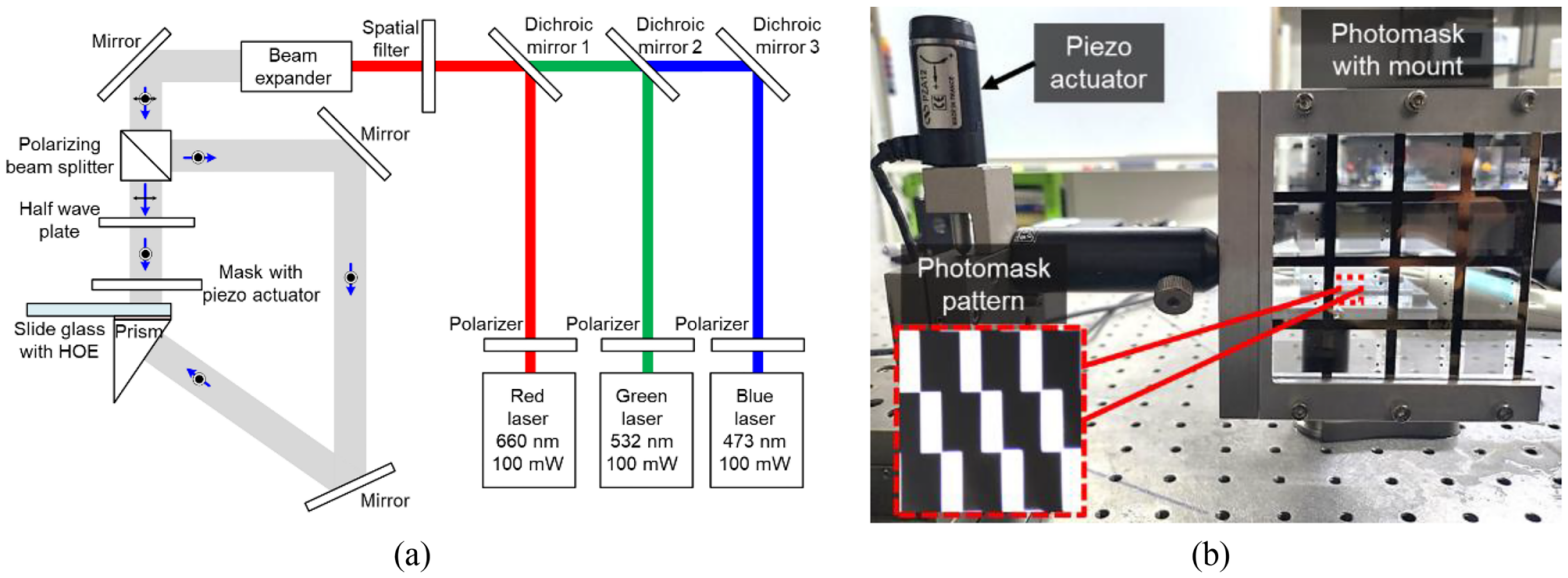
Experimental setup for the implementation of SMDG HOEs by using a photomask and three laser sources. (**a**) Dichroic mirrors 1, 2, and 3 only reflect light in the R, G, and B wavelength bands, respectively. (**b**) Photograph of the photomask alignment setup and close-up of the photomask pattern within the red dotted box.

Figure 1a depicts a schematic of the experimental apparatus designed to record an HOE with an SMDG pattern mask. A diode-pumped laser operating and emitting at wavelengths of 660 nm, 532 nm, and 473 nm functions as the light source. The exposure power and time were set to 1 mW and 20 s to achieve the saturation of diffraction efficiency. We used a spatial filter of solely a pinhole to enhance the spatial profile of the laser beam by removing random intensity fluctuations. To keep the collimation without divergence, diameter of the pinhole was employed 50 μm, which is much larger than the wavelength. Subsequently, a polarization beam splitter was implemented along with a half-wave plate to align the polarization directions of both the beams with s-polarization. The photopolymer substrate was then affixed to a slide glass. A prism was placed along the path of the reference beam, and glycerin, possessing a refractive index of 1.44, was used as an index-matching medium to eliminate the air gap between the prism and the photopolymer. As shown in Fig. 1b, to spatially modulate the HOE, a photomask was positioned such that the recording of each specific wavelength was selectively allowed. To minimize the diffraction effect by photomask pattern, we minimize the gap between photomask and HOE by finely tuning through a piezo actuator. The red dotted box is the magnified photomask, which is the size in micrometers. After the SMDG HOE was recorded, the glycerin used as an index-matching fluid was completely removed to ensure that there was no distortion or degradation of the display quality.

**Figure 2:**
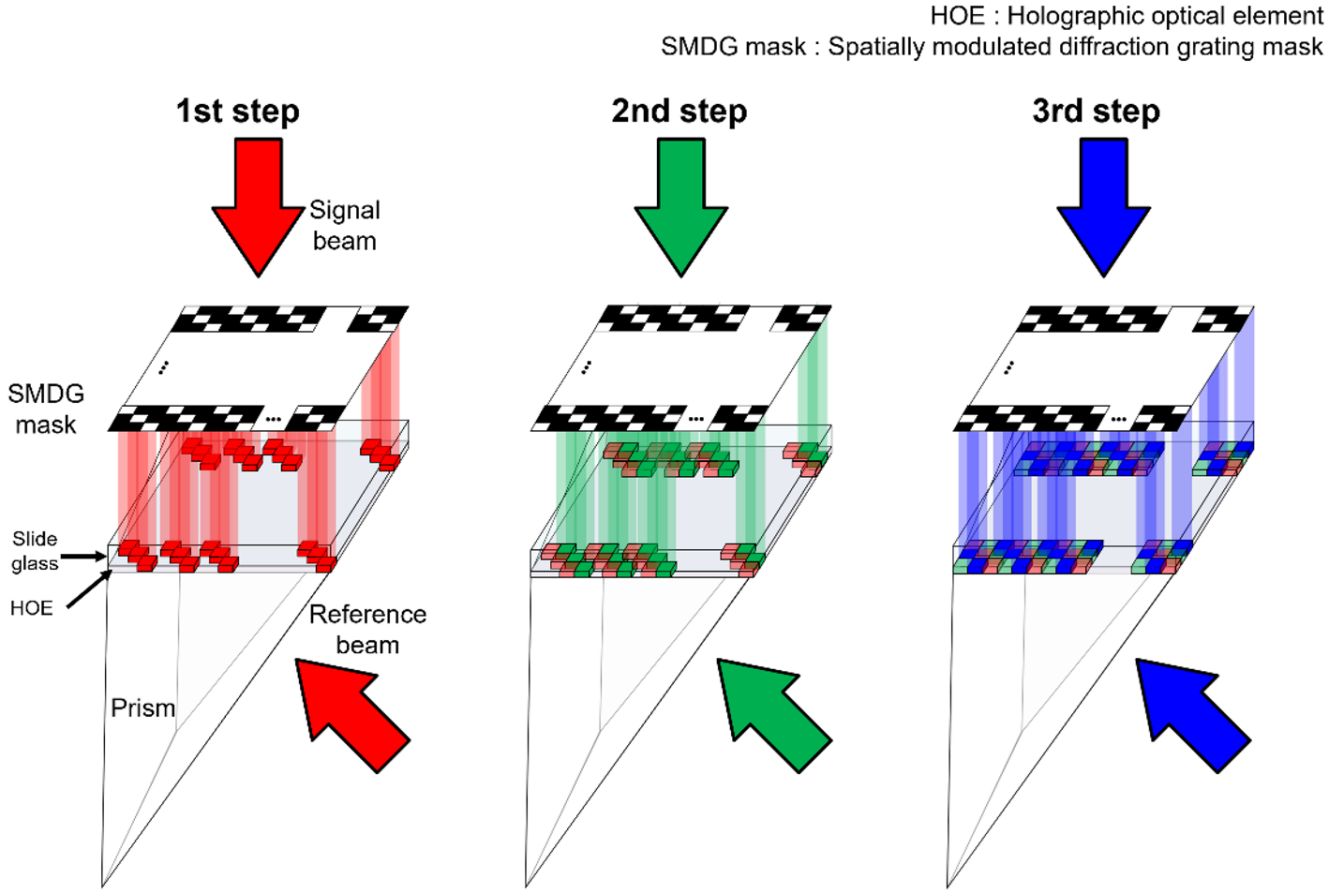
Conceptual diagram illustrating the recording process of the SMDG pattern on the HOE. The R, G, and B light beams were recorded using their respective masks.

Figure 2 depicts the conceptual process of the mask alignment, which is used to demonstrate the SMDG HOE recording of the three laser sources. The experimental procedure was conducted in a series of steps. Initially, the gap between the SMDG pattern mask and the HOE was minimized using a piezo actuator. After that, the photopolymer was exposed to an R light beam only in the corresponding, and all the other subpixels were occluded by using the chrome coating within SMDG mask. Only the light transmitted through the mask interferes with the reference beam to form the desired pattern. Subsequently, a G pattern was inscribed into a separate region of the photopolymer by actuating the nanoactuator. A similar process was repeated for light B in the final step. This methodology was used to successfully record an SMDG pattern on a single HOE, facilitating the diffraction of R, G, and B light, thereby broadening the color gamut. After exposure to the R, G, and B light beams, a halogen lamp was illuminated for 10 min to fix the recorded HOE.

### Design of SMDG pattern

**Figure 3:**
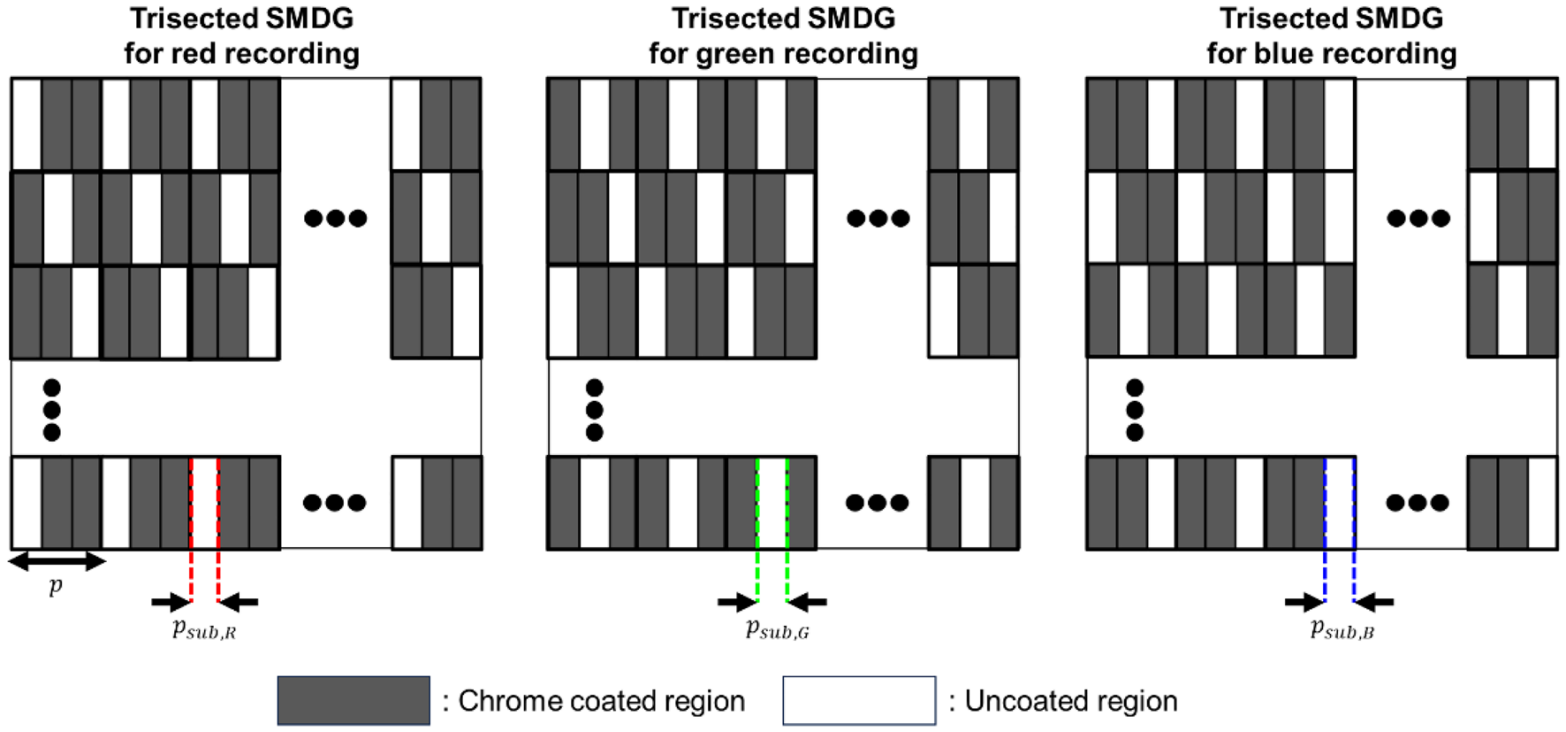
Mask pattern for the trisected version of the SMDG. Black regions represent chrome regions to block the beam, while white regions denote uncoated regions to transmit the beam.

Several factors must be considered in the design of an SMDG pattern: the pixel pitch or pixels per inch (PPI), optical characteristics of the HOE, and spectral response of either the human eye or the camera. Contrary to conventional display devices such as smartphones, tablets, and laptops, HMDs require an exceptionally high PPI density owing to their proximity to the human eye. An insufficient PPI density can result in a visual artifact known as the screen door effect, wherein the fine lines separating the subpixels or pixels become perceptible, thereby diminishing the immersive experience. Typically, HMDs require a PPI exceeding 2000, implying that the dimensions of a single pixel must not exceed 12.7 μm, to prevent the screen door effect^27,28^. To mitigate the screen door effect, we configured the subpixel pitches (*p*_*sub,R*_, *p*_*sub,G*,_ and *p*_*sub,B*_) to be 3 μm. Consequently, the total pixel pitch (*p*) is 9 μm, equal to the combined width of the three subpixel pitches, while the height of the pixel is set to 9 μm, aligning with the total pixel pitch. Given the recording beam size of 1.5 cm, the total resolution of recorded pixels within the HOE is approximately 1666 × 1666, which surpasses the resolution of the beam projector to prevent any reduction in image quality. The corresponding SMDG pattern within the HOE can be implemented through the customized design of the photomask, which is tailored to match the subpixel pitches. Figure 3 depicts the trisected version of the photomask pattern with the same subpixel pitch. Within the mask pattern, the black regions represent the chrome-coated regions and the white regions denote the uncoated regions. Spatial modulation is implemented by these mask patterns, penetrating only the uncoated regions, and is obstructed by the coated regions. The transmitted light then interacts with the photopolymer and interferes with the reference beam, enabling pattern recording. Using this methodology, three lights (R, G, and B) were recorded on a single HOE through spatial modulation.

**Figure 4:**
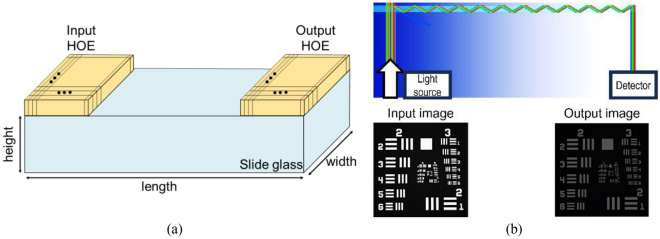
(**a**) Schematic of a WG display that employs an SMDG HOE constructed on slide glass and photopolymer. (**b**) Ray tracing figure based on trisected HOE-based WG display and input image (USAF 1951 target) and output image observed by the detector through the imaging simulation.

Figure 4a presents a schematic of the WG display, where the photopolymer films were affixed to both the ends of a slide glass measuring 26 mm in width, 76 mm in length, and 1 mm in height. Figure 4b depicts the outcomes when incident parallel light of R, G, and B wavelengths impinged on the WG guided by the SMDG-configured HOE. We performed ray-tracing analysis using Zemax software, and confirmed that the paths followed by the R, G, and B rays were congruent and propagated along the same direction. This simulation represents a WG display employing the trisected version of the HOE, where the observed loss is attributed to the dispersion of the R, G, and B wavelengths across individual unit pixels, each accounting for only one-third of the spectrum. We also performed an image simulation by projecting the input image (USAF 1951 target), as shown in Fig. 4b, yielded the observation that R, G, and B components of the input image were faithfully transmitted with only a one-third reduction in the light intensity. This loss is caused by the trisected structure within the HOE, which can be attributed to the subdivision of the respective subpixel areas, resulting in the diffraction of the R, G, and B beams within each unit pixel in a ratio of 1/3.

**Figure 5:**
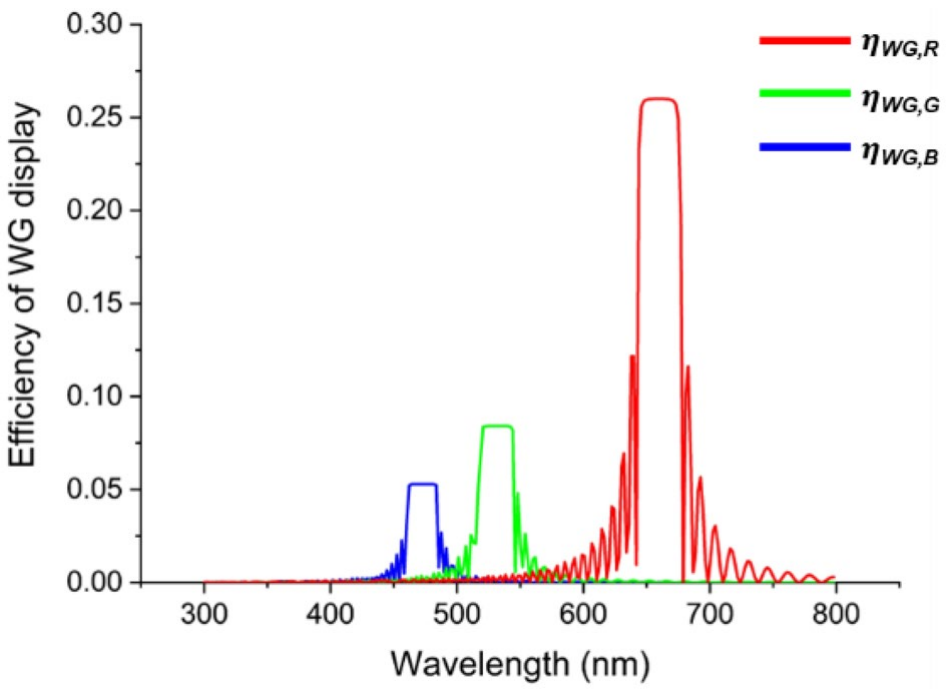
Efficiency spectrum of a trisected HOE-based WG display in the visible wavelength band.

HOEs typically exhibit wavelength-dependent properties, where the transmittance and absorption vary with the wavelength, which necessitates due consideration. To consider this point, we conducted a simulation with the same setup as that shown in Fig. 4a and applied the optical characteristics of the photopolymer (HX200, Geola) employed in this study^24,29^. Figure 5 depicts the efficiency spectra which is the ratio of input and output beam intensity. Evidently, the efficiencies across the R, G, and B spectral bands are non-uniform owing to the inherent optical properties of the HOE, with the R band presenting relatively high efficiency and the B band presenting lower efficiency.

The efficiencies of the trisected HOE-based WG display over the three wavelength bands (${\eta }_{WG,tri,B}$, ${\eta }_{WG,tri,G}$, and ${\eta }_{WG,tri,R}$) can be expressed by Eqs. (1–3) and intensity of the trisected type WG output (${I}_{WG,tri}\left(\lambda \right)$) corresponding to the input intensity (${I}_{in}\left(\lambda \right)$) is depicted in Eq. (4).

1 $${\eta }_{WG,tri,B}\left(\lambda \right)={\eta }_{WG,B}\left(\lambda \right)\times \frac{{p}_{sub,B}}{p}, \left(450 nm<\lambda <490 nm\right).$$

2 $${\eta }_{WG,tri,G}\left(\lambda \right)={\eta }_{WG,G}\left(\lambda \right)\times \frac{{p}_{sub,G}}{p},\left(530 nm<\lambda <590 nm\right).$$

3 $${\eta }_{WG,tri,R}\left(\lambda \right)={\eta }_{WG,R}\left(\lambda \right)\times \frac{{p}_{sub,R}}{p},\left(630 nm<\lambda <700 nm\right)$$

4 $${I}_{WG,tri}\left(\lambda \right)={\eta }_{WG,tri }\left(\lambda \right)\times {I}_{in}\left(\lambda \right).$$

where, ${\eta }_{WG,B}\left(\lambda \right)$, ${\eta }_{WG,G}\left(\lambda \right)$, ${\eta }_{WG,R}\left(\lambda \right)$ represents the efficiency of each general HOE-based WG display, individually recorded with a single B, G, or R wavelength laser. ${\eta }_{WG,tri}(\lambda )$ denotes the diffraction efficiency of the trisected HOE-based WG display within the visible light wavelength range, including ${\eta }_{WG,tri,B}$, ${\eta }_{WG,tri,G}$, and ${\eta }_{WG,tri,R}$.

**Figure 6:**
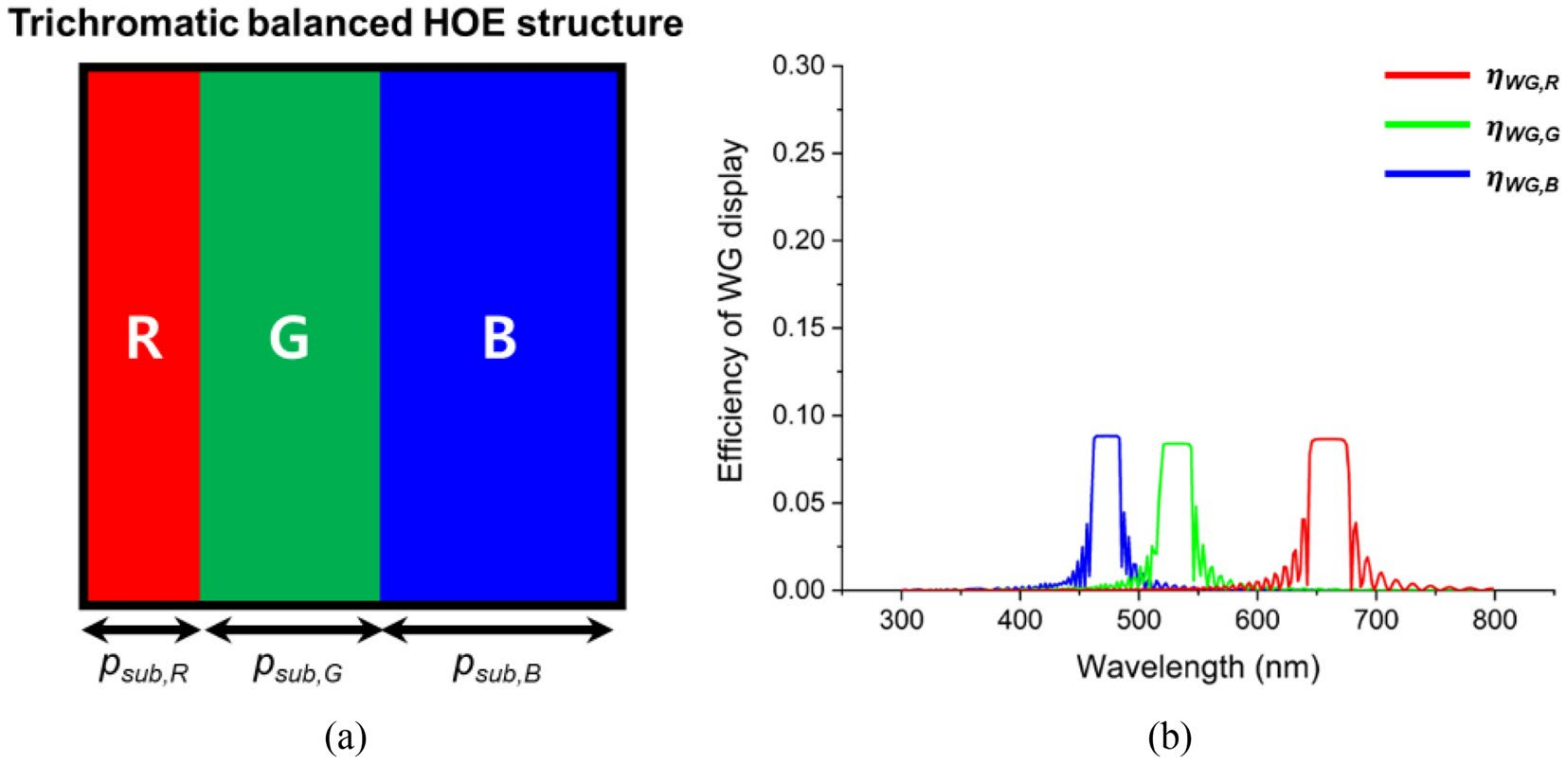
(**a**) Conceptual diagram of a single pixel recorded using the TCB-HOE mask pattern. It considers the optical characteristics of the HOE. (**b**) Efficiency spectrum of the TCB-HOE-based WG display.

The non-uniformity among these efficiencies will result in a narrowed color gamut, which limits the color representation range of the display system. We compensated for the efficiency difference among the three bands by adjusting the subpixel pitch dimensions (*p*_*sub,R*_, *p*_*sub,G,*_* p*_*sub,B*_) within the unit pixel. Given that the efficiencies of the R, G, and B bands were approximately 5.3%, 8.4%, and 26.0%, respectively, we designed a TCB-HOE, as shown in Fig. 6a. While maintaining a unit pixel pitch (*p*), we set *p*_*sub,R*_ to 2 μm, and increased *p*_*sub,G*_ and *p*_*sub,B*_ to 3 μm and 4 μm, respectively. Figure 6b depicts the simulated efficiencies of the WG display based on the TCB-HOE. The efficiencies across the R, G, and B bands were remarkably uniform at 8.4%, 8.7%, and 8.8%, respectively. This indicates that the disparities between the three band efficiencies were significantly reduced by fine-tuning the subpixel pitches. Consequently, the detector receives the output of the WG display as a superposition of the three uniform-band spectra, thereby enhancing the overall color reproduction.

Another pivotal factor is the spectral sensitivity of the receiving device, whether it is a human eye or a camera. In humans, the three types of cone cells that perceive R, G, and B wavelengths are termed short (S), medium (M), and long (L) cones, respectively^30^. Each type of cone is sensitive to a particular wavelength band and possesses unique sensitivity levels, forming what is known as a tristimulus response. Therefore, the human eye may perceive light with the same intensity as having different intensities. This must be considered during the color gamut optimization phase. In this study, we focused on the spectral response of a CCD camera (BFLY-U3-50H5C-C, FLIR) rather than the human eye to demonstrate the potential for color gamut expansion.

**Figure 7:**
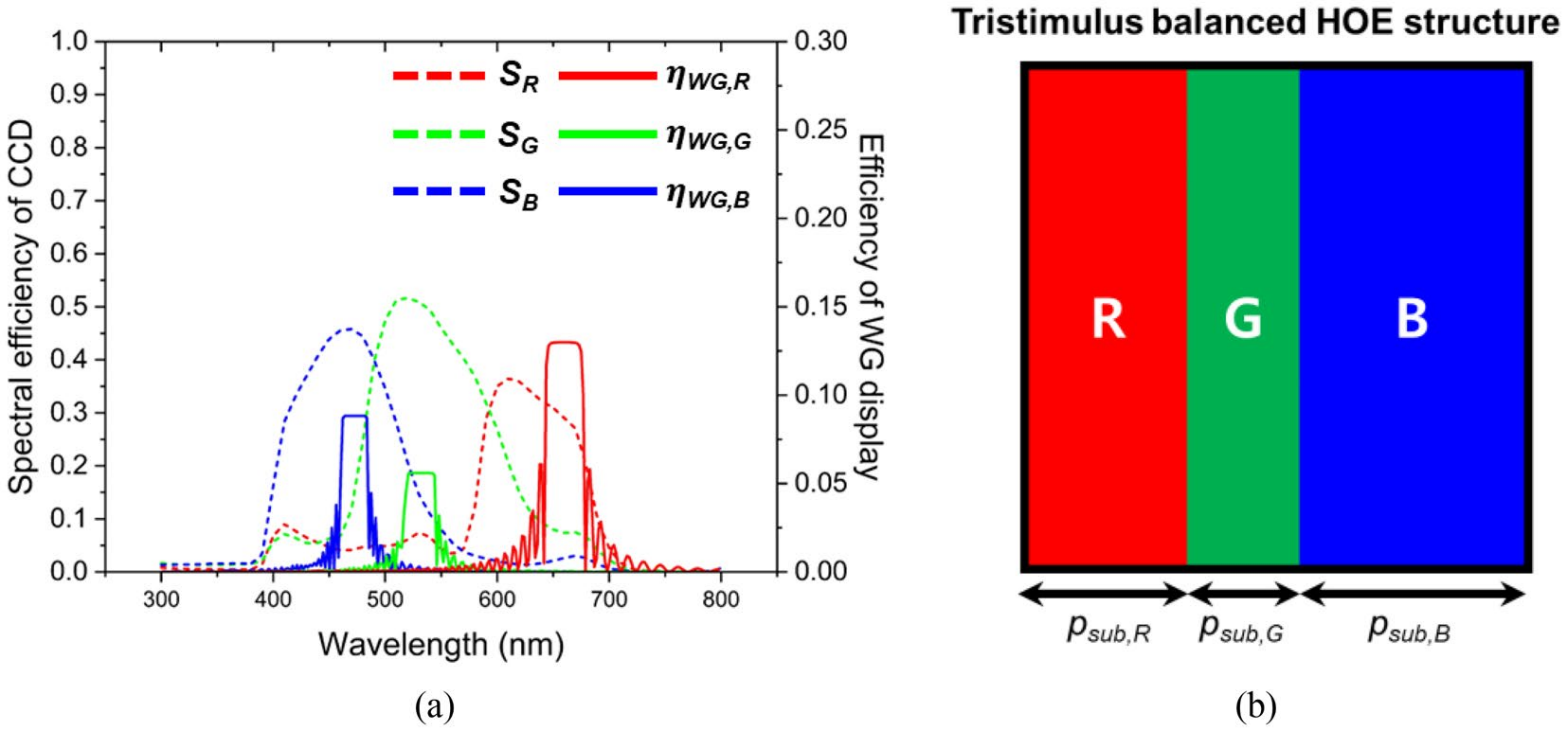
(**a**) Spectral sensitivity of the CCD camera employed (dotted line, left axis), with high sensitivity to the G band and low sensitivity to the R band and efficiency of WG display based on TSB-HOE (solid line, right axis). (**b**) Conceptual diagram of a single pixel recorded using the TSB-HOE mask pattern. It considers the optical characteristics of the HOE and the spectral response of the CCD camera.

Figure 7a depicts the spectral response of the employed CCD camera (represented by *S*_*R*_(*λ*), *S*_*G*_(*λ*), and *S*_*B*_(*λ*) as a dotted line), which demonstrates that the maximum sensitivity to the B, G, and R bands is approximately 44%, 52%, and 37%, respectively. The color perception of a CCD camera from the light transmitted through a WG display can be expressed mathematically by using Eqs. (5–7). These equations represent the intersection between the corresponding tristimulus functions (dashed lines, left axis) and the efficiency spectra of the WG display output (solid lines, right axis), as shown in Fig. 7a. Since the intensity of the output from the WG display (*I*_*WG,R,G,B*_ (*λ*)) for each wavelength band can be tuned by adjusting the subpixels, it is also feasible to optimize the perception level, *B* (*λ*), *G* (*λ*), *R* (*λ*), which indicates the degree to which the CCD perceives the B, G, and R values, respectively. A uniform ratio of these values corresponds to a broader color gamut, thereby expanding the range of expressible colors. We designed the TSB-HOE with subpixel pitches (*p*_*sub,R*_, *p*_*sub,G,*_* p*_*sub,B*_) set at 3 μm, 2 μm, and 4 μm to optimize this equilibrium, as shown in Fig. 7b.

5 $$B\left(\lambda \right)={\int }_{450 nm}^{490 nm}{I}_{WG}\left(\lambda \right)\times {S}_{B}\left(\lambda \right)d\lambda$$

6 $$G\left(\lambda \right)={\int }_{530 nm}^{590 nm}{I}_{WG}\left(\lambda \right)\times {S}_{G}\left(\lambda \right)d\lambda .$$

7 $$R\left(\lambda \right)={\int }_{630 nm}^{700 nm}{I}_{WG}\left(\lambda \right)\times {S}_{R}\left(\lambda \right)d\lambda$$

Table 1 presents a comparative analysis of the B, G, and R values as perceived by the CCD camera across three distinct SMDG configurations: trisected, TCB, and TSB-type HOE. All three types of the B, G, and R values were derived by using Eqs. (5–7) and then normalized by setting B to 1. The data revealed that the TSB-HOE achieved a uniform ratio of B, G, and R values, indicating comparable sensitivity across the R, G, and B wavelength bands. Evidently, the WG display based on the TSB-HOE presents the most enhanced color gamut and exhibits excellent color reproduction.

**Table 1:** Comparative assessment of the perception level in WG display based on three types SMDG HOEs.

Type	B	G	R
Trisected HOE	1	1.47	1.10
Trichromatic-balanced-HOE (TCB-HOE)	1	1.34	0.76
Tristimulus-balanced-HOE (TSB-HOE)	1	1.02	1.04

## Results

**Figure 8:**
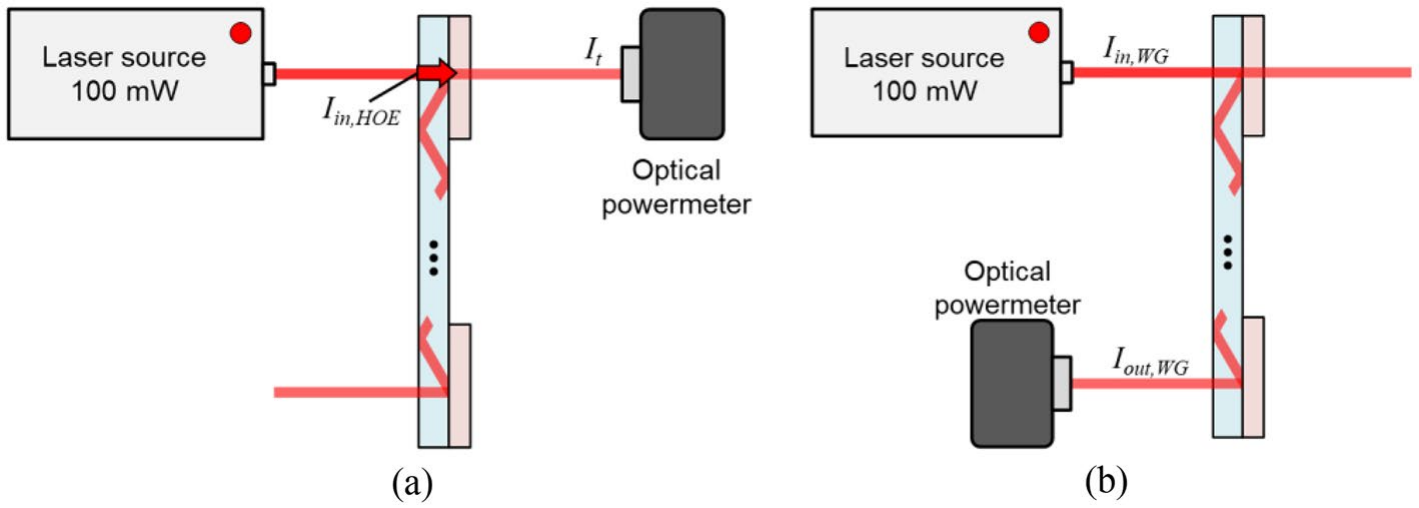
Schematic of the experimental setup used to measure the efficiency of the (**a**) diffraction and (**b**) WG displays. The diffraction efficiency was calculated by using Eqs. (8) and (9), which involve the input beam intensity (*I*_*in,HOE*_) passing through the slide glass and the transmitted intensity (*I*_*t*_). The WG display efficiency was defined as the measured ratio of the output intensity (*I*_*out,WG*_) to the input intensity of the WG display (*I*_*in,WG*_), as expressed in Eq. (10).

To assess the wavelength-specific efficiency and potential color gamut extension of the three SMDG HOE types, we established an experimental setup. Figure 8a,b illustrates the arrangement for measuring the diffraction efficiency of the SMDG HOEs and the WG efficiency, respectively. To ascertain this diffracted beam intensity, we measured the intensities of both the input and transmitted beams by using an optical powermeter (918D-SL-OD3R, Newport). Furthermore, the optical powermeter was placed at the observer's location to measure the output power (*I*_*out*_ (*λ*)) for each type of WG display. The diffraction intensity and efficiency (*I*_*d*_ (*λ*) and ${\eta }_{(B,G,R)}$ (*λ*)) were calculated by using Eqs. (8–9). These equations incorporate the input beam intensity for the HOE (*I*_*in,HOE*_ (*λ*)) passing through the slide glass, as well as the transmitted intensity (*I*_*t*_ (*λ*)). The WG display efficiency was defined as the measured ratio of the output intensity (*I*_*out,WG*_ (*λ*)) to the input intensity of the WG display (*I*_*in,WG*_ (*λ*)), given by Eq. (10).

8 $${I}_{d}(\lambda )={I}_{in,HOE}(\lambda )-{I}_{t}(\lambda )$$

9 $${\eta }_{(B,G,R)}=\frac{{I}_{d}(\lambda )}{{I}_{in,HOE} (\lambda )}\times 100$$

10 $${\eta }_{WG,(B,G,R)}=\frac{{I}_{out,WG}(\lambda )}{{I}_{in,WG}(\lambda )}\times 100$$

Here, ${\eta }_{(B,G,R)}$ and ${\eta }_{WG,(B,G,R)}$ represent the individual efficiency of diffraction and WG display for the B, G, and R wavelengths. The input light source comprised a single-frequency laser with wavelengths of 660 nm, 532 nm, and 473 nm for R, G, and B, respectively.

Based on this setup and by using Eqs. (8–10), Table 2 presents the measured diffraction and waveguide efficiencies. Since spatial modulation involves the subdivision of a single pixel, inherent losses occur in all three types of HOEs. The trisected HOE, which comprises a structure divided by a pixel into thirds, exhibits a significant deviation in the diffraction efficiency among the three spectral bands. This is primarily attributed to the characteristics of the HOE. The TCB-HOEs, optimized to achieve a consistent output power without considering the receiver sensitivity, exhibited a relatively uniform diffraction efficiency. This uniformity is attributed to the uniform sensitivity of the optical power meter across the three wavelength bands, unlike that of the CCD camera. Conversely, the TSB-HOE presents a relatively non-uniform efficiency since it considers the spectral sensitivity of the CCD camera. The AR-HMD is supposed to provide a wide color gamut to the user, unlike an optical powermeter.

**Table 2:** Comparative analysis to measure the efficiency of diffraction and WG display based on three different HOE-type SMDGs.

Type	${\eta }_{B}$ (%)	${\eta }_{G}$ (%)	${\eta }_{R}$ (%)	${\eta }_{WG,B}$ (%)	${\eta }_{WG,G}$ (%)	${\eta }_{WG,R}$ (%)
Trisected HOE	22.1	23.8	24.4	4.4	5.2	5.4
Trichromatic-balanced-HOE (TCB-HOE)	23.7	24.6	23.4	5.1	5.5	4.9
Tristimulus-balanced-HOE (TSB-HOE)	26.0	24.0	29.0	6.2	5.2	7.7

**Figure 9:**
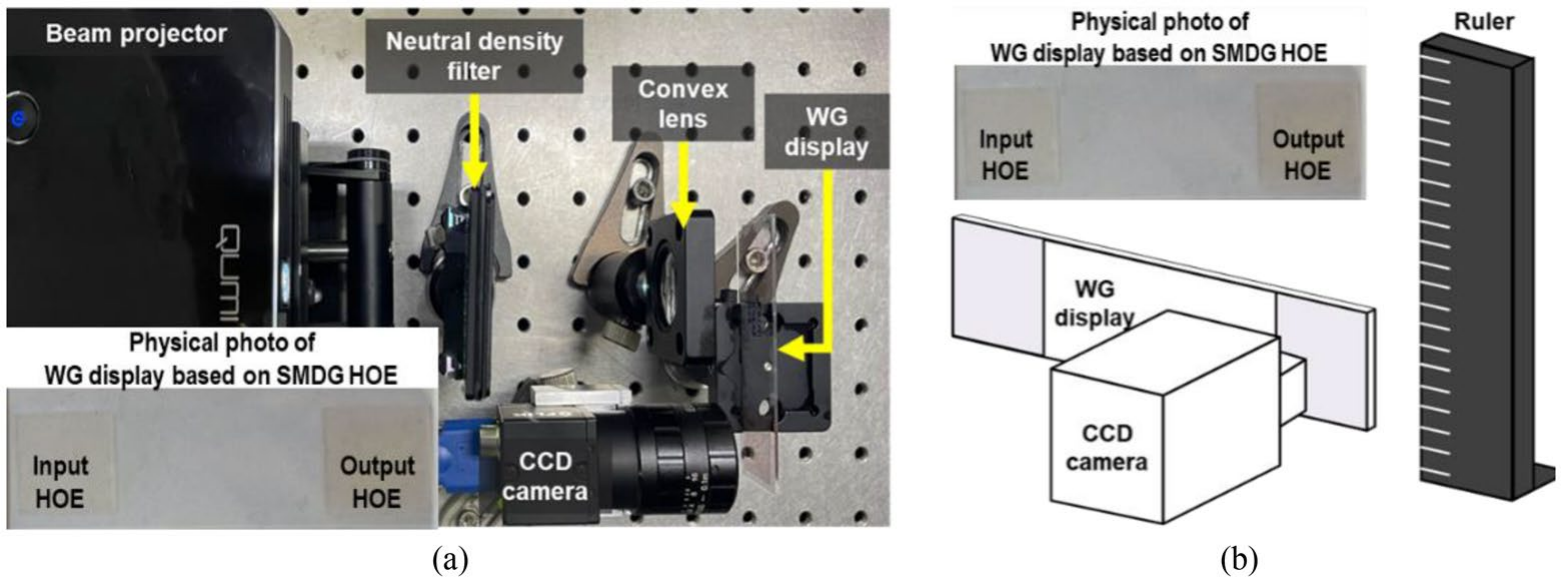
(**a**) Experimental setup for capturing the WG display output. The beam projector’s virtual content was resized and dimmed by using a neutral-density filter and convex lens before being confined to the WG display, and the output image is captured by the CCD camera. (**b**) Physical photograph of WG display based on the SMDG HOE and conceptual scheme to demonstrate AR with a ruler functioning as a physical object.

To show the applicability of the three aforementioned HOE-based WG displays for AR-HMDs, an experimental setup was established, as shown in Fig. 9a. The virtual content emanating from the beam projector was subjected to brightness attenuation via a neutral-density filter. The beam was collimated and resized to the appropriate dimensions of approximately 3.5 mm by using a 1-inch convex lens. Subsequently, the virtual content was delivered to the CCD camera through a WG display. Figure 9b presents a schematic of the physical object (ruler), WG display, and CCD camera, which facilitates the AR implementation. In AR demonstration, the ruler was used as a physical object.

**Figure 10:**
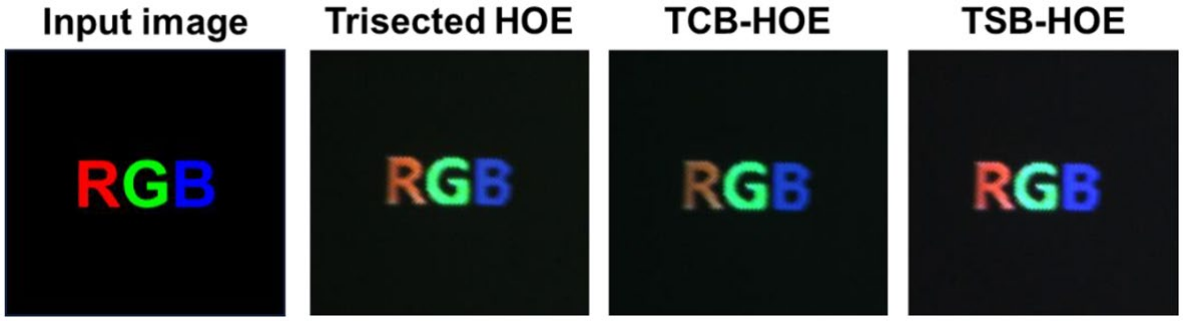
Captured experimental result image through the CCD camera after delivery via the three different types of SMDG HOEs-based WG displays. The input image (labeled RGB) served as the input for the beam projector.

Prior to the AR implementation, Fig. 10a–d present the results of the AR experiments in a darkroom. The original image (labeled RGB) served as the input for the beam projector, while Fig. 10b–d depict the images captured through the CCD camera after transmission via the three different types of WG displays. The field of view (FOV) is defined as the angle at which the user can view the virtual content. It was calculated according to the size of the virtual content and the distance from the CCD camera to the focal point of the virtual content. The measured FOV was approximately 8°. This relatively narrow FOV was due to the narrow angular selectivity inherent to the HOE-based WG display. The virtual content in R, G, and B was delivered for all three configurations; however, there was a difference in the brightness of the three colors. In the trisected version, G exhibits relatively higher brightness, whereas B appears relatively darker. With the TCB-HOE, G appears to be brighter, and R appears to be less luminous. Conversely, TSB-HOE yields a uniform and bright output, with all three "RGB" letters being distinctly vibrant.

**Figure 11:**
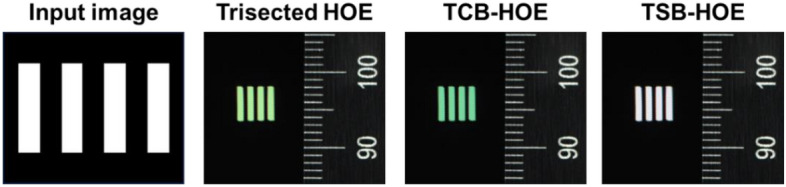
Experimental results of the three different types of SMDG HOE-based WG displays. Four white lines were adopted as the input image to quantitatively analyze the three colors detected by the CCD camera. The overlap of the physical object (the ruler) with virtual content (the white lines) showed that AR was implemented.

**Figure 12:**
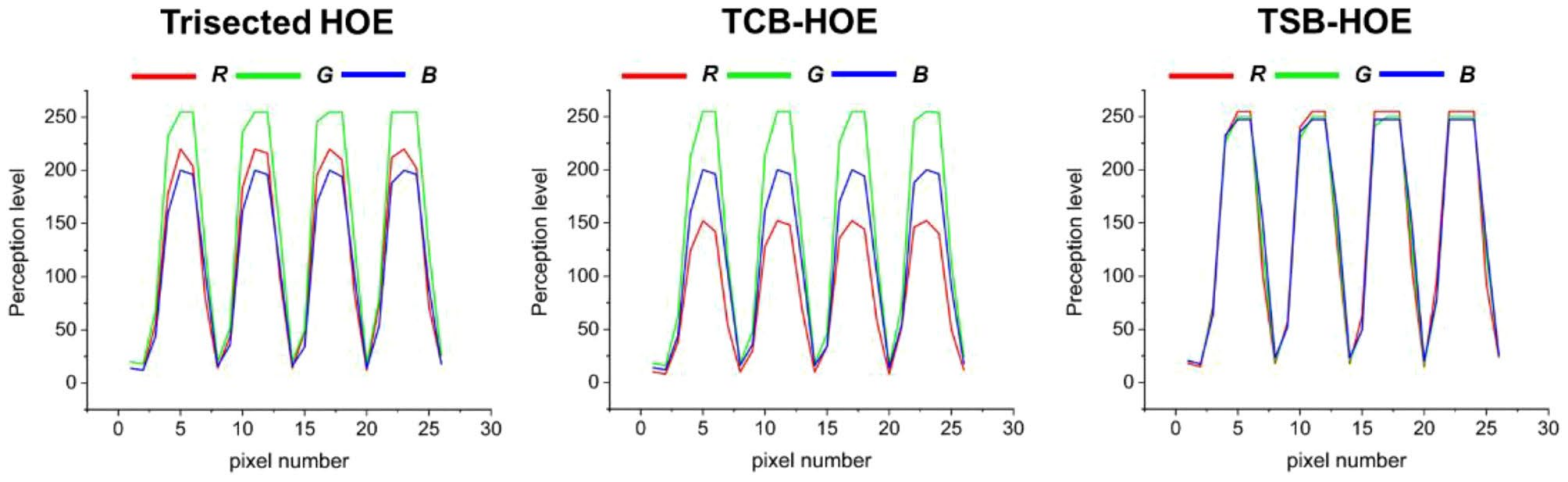
The R, G, and B intensity data for pixels extracted along the horizontal axis of the virtual content part in the three types of WG display results presented in Fig. 11.

**Figure 13:**
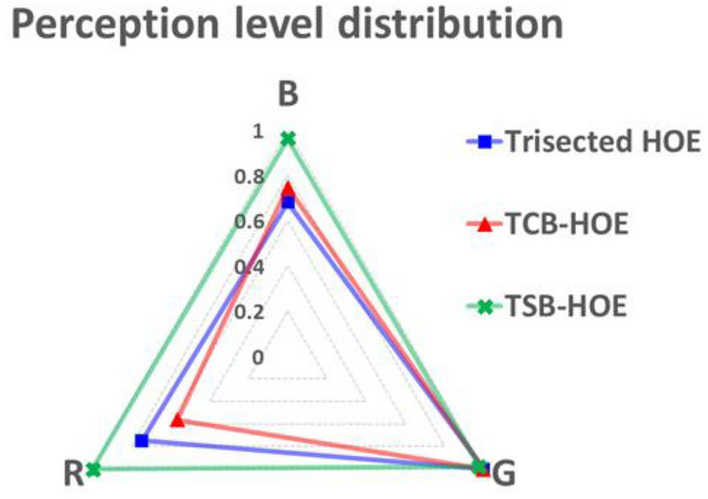
Perception level distribution chart for the CCD camera on the three types SMDG HOE-based WG display by applying the measured efficiencies to Eqs. (5–7).

Furthermore, we adopted white lines as an input image to quantify the perception levels of R, G, and B detected by the CCD camera. Figure 11 illustrates the successful implementation of AR by overlapping a physical object (the ruler) with virtual content (the white lines). The trisected type HOE exhibited a yellowish tint instead of a white tint, primarily because of the excessive dominance of R and G over B. In contrast, the TCB-HOE output leaned toward a blue-green hue, reflecting an overemphasis on G and B relative to R. On the other hand, the TSB-HOE configuration effectively exhibited a true white color. For the quantitative analysis, we extracted the horizontal rows of white lines through MATLAB to measure the intensities of the R, G, and B colors, as shown in Fig. 12. As an intuitive results, the trisected types depicted a disproportionate predominance of R and G when compared to B. Conversely, the TCB-HOE output predominantly comprised B and G, reflecting an overemphasis on G and B relative to R. However, in the TSB-HOE configuration, the B, G, and R values were observed to be uniformly distributed. Figure 13 shows the perception level distribution of the CCD camera on the three types SMDG HOEs, which is similar to the chromaticity diagram. It was obtained by applying the measured efficiency of each type WG display to the Eqs. (5–7) and normalizing the calculated level. Among them, TSB-HOE is uniformly distributed to the three primary color owing to the optimization of the subpixel pitches to the CCD’s spectral response. With the SMDG HOE-based WG display, color in the range formed by R, G, and B vertices of each type can be displayed by adjusting the R, G, and B ratio of the input beam.

## Discussion

In this study, we devised a novel method that uses spatially modulated diffractive optical elements to extend the color gamut of WG displays. We demonstrated that the balance between the R, G, and B intensities can be fine-tuned by modulating the subpixel pitch through simulations and experimental analysis conducted using the HOE. A comparative analysis of the trisected, TCB, and TSB-HOE configurations highlights the significance of taking into account both the spectral sensitivity of the observer (the CCD camera used in this study) and the intrinsic characteristics of the HOE to achieve optimization. We also demonstrated that the proposed technique is versatile for this optimization.

We elucidated the construction of the SMDG HOE and confirmed that the R, G, and B wavelengths propagated co-directionally, which was verified through ray tracing and imaging simulations performed using Zemax in Section "Methods". Additionally, we describe the most important factors affecting the expansion of the color gamut and explain the theoretical basis for determining the SMDG subpixel pitch. The process of designing TCB and TSB-HOEs using the diffraction efficiencies in the R, G, and B bands obtained through simulations is described sequentially. Furthermore, Table 2 compares the R, G, and B color balances perceived by the CCD camera in the context of three different SMDG HOE-based WG displays.

We also experimentally evaluated (Section "Results") the diffraction efficiency and WG display efficiency for the R, G, and B wavelengths for the three HOE types. The measurements are summarized in Table 2. Subsequently, we demonstrated the AR capabilities to validate the applicability of the proposed technique for AR-HMD optics. The effectiveness of the SMDG concept is further substantiated by using a color-filtered graph extracted through MATLAB. Among the SMDG types that were considered, the TSB-HOE-based WG display, which was optimized corresponding to both the HOE characteristics and spectral sensitivity of the observing CCD camera, exhibited the most well-balanced tricolors.

The proposed method presents several advantages over the techniques introduced in previous studies. Primarily, it facilitates a more precise control over the tristimulus values of the displayed colors by enabling independent adjustments to the pitch of each pixel. This granular control is particularly beneficial when configuring the display to the spectral sensitivities of diverse receivers such as CCD cameras or the human visual system. Secondly, the proposed approach presents high reproducibility and reliability. Prior approaches, which depend on layering the HOEs and modulating exposure times for individual colors, could not effectively optimize the tristimulus values since they did not consider spectral sensitivity, a crucial factor that was addressed in this study. Additionally, these methods are constrained by their limited reproducibility and reliability, since optimization relies only on the experimentally obtained data^25,26^.

Although our method offers several advantages, it also faces certain limitations. There are inherent losses in the SMDG structure when unrecorded light is incident on the subpixels. An illustrative example is when G or B light enters a subpixel originally recorded by R. However, a certain degree of loss is inevitable for a diffraction-grating-based WG display to diffract the three colors of light. The losses outlined in this paper are comparable to those encountered in the previously proposed methods^24–26^. Additionally, the mask pattern employed in this paper was produced through a photolithography method, with a minimum line width of 1 μm, thereby imposing a certain limitation on achieving optimization. Notably, the meticulous adjustment of the mask represents a labor-intensive and intricate process, and the pattern-recording procedure necessitates a high degree of precision. To address these challenges, we aim to implement the SMDG pattern recording through semiconductor processing techniques that can achieve ultra-fine processing. More elaborate patterns than those showcased by the SMDG pattern presented in this paper can be achieved by utilizing advanced techniques, such as e-beam lithography; this will be explored in future studies. It is crucial to conduct a thorough analysis of secondary effects, such as diffraction, that arise from reducing the subpixel pitch, to ensure accurate and good HOE performance. Despite this challenge, we firmly believe that the proposed technique presented holds significant potential for notably enhancing the color gamut of HOE-based WG displays.

## Conclusion

In this study, we introduce a novel method that employing spatial modulation method to enhance the color gamut of HOE-based WG displays. The utility of SMDG methods lies in its capacity to diffract R, G, and B rays co-directionally, thereby enabling an immersive full-color display. The underlying principles and theoretical foundations of the SMDG are elaborated through Zemax simulations and theoretical analyses. The experimental analysis demonstrates that the proposed method herein yields a significant improvement in the color gamut of HOE-based WG displays. The contributions of this study to the field of HOE-based WG displays are two-fold. First, we introduce a novel and effective approach to extend the range of the representable colors, thereby expanding the color gamut. Second, the proposed method outperformed its predecessors in terms of both the reproducibility and reliability. The findings of this study will potentially lead to significant advancements in the development of HOE-based WG displays.

## Data Availability

The data to support the results within this paper is available from the corresponding author on reasonable request.

## References

[1] Jeong J (2020). Holographically printed freeform mirror array for augmented reality near-eye display. IEEE Photon. Technol. Lett..

[2] Billinghurst M, Clark A, Lee G (2014). A survey of augmented reality. Found. Trends. Hum. -Comput. Interact..

[3] Wang YJ, Chen PJ, Liang X, Lin YH (2017). Augmented reality with image registration, vision correction and sunlight readability via liquid crystal devices. Sci. Rep..

[4] Lee S (2019). Tomographic near-eye displays. Nat. Commun..

[5] Jang C (2017). Retinal 3D: Augmented reality near-eye display via pupil-tracked light field projection on retina. ACM Trans. Graph..

[6] Xiong J (2021). Augmented reality and virtual reality displays: Emerging technologies and future perspectives. Light Sci. Appl..

[7] Cheng D, Wang Y, Hua H, Talha MM (2009). Design of an optical see-through head-mounted display with a low f-number and large field of view using a freeform prism. Appl. Opt..

[8] Zheng Z, Liu X, Li H, Xu L (2010). Design and fabrication of an off-axis see-through head-mounted display with an x-y polynomial surface. Appl. Opt..

[9] Cheng D, Wang Y, Hua H, Sasian J (2011). Design of a wide-angle, lightweight head-mounted display using free-form optics tiling. Opt. Lett..

[10] Mukawa H (2009). A full-color eyewear display using planar waveguides with reflection volume holograms. J. Soc. Inf. Disp..

[11] Zhang Y, Fang F (2019). Development of planar diffractive waveguides in optical see-through head-mounted displays. Precis. Eng..

[12] Peng H (2014). Design and fabrication of a holographic head-up display with asymmetric field of view. Appl. Opt..

[13] Cheng D (2021). Design and manufacture AR head-mounted displays: A review and outlook. Light Adv. Manuf..

[14] Xiao J, Liu J, Han J, Wang Y (2019). Design of achromatic surface microstructure for near-eye display with diffractive waveguide. Opt. Commun..

[15] Guo LJ (2007). Nanoimprint lithography: Methods and material requirements. Adv. Mater..

[16] Miller JM, De Beaucoudrey N, Chavel P, Cambril E, Launois H (1996). Synthesis of a subwavelength-pulse-width spatially modulated array illuminator for 0.633 mm. Opt. Lett..

[17] Lim Y (2021). Holography, fourier optics, and beyond photonic crystals: Holographic fabrications for Weyl points, bound states in the continuum, and exceptional points. Adv. Photonics Res..

[18] Vorzobova, N. & Sokolov, P. Application of photopolymer materials in holographic technologies. *Polymers* (Basel) 11 (2019). 10.3390/polym11122020 PMC6960734 31817649

[19] Xiong J, Yin K, Li K, Wu S-T (2021). Holographic optical elements for augmented reality: Principles, present status, and future perspectives. Adv. Photonics Res..

[20] Lee Y.-H, Yin K, Wu S-T (2017). Reflective polarization volume gratings for high efficiency waveguide-coupling augmented reality displays. Opt Express.

[21] Weng Y, Xu D, Zhang Y, Li X, Wu S-T (2016). Polarization volume grating with high efficiency and large diffraction angle. Opt Express.

[22] Gu, Y. *et al.* Holographic Waveguide Display with Large Field of View and High Light Efficiency Based on Polarized Volume Holographic Grating. *IEEE Photonics J ***14**, (2022).

[23] Weng, Y. *et al.* Liquid-crystal-based polarization volume grating applied for full-color waveguide displays. *Opt Lett* **43**, 5773 (2018). 10.1364/OL.43.005773 30499990

[24] Wu HY (2022). Time-scheduled exposure method for full-color high diffraction efficiency and uniformity of a photopolymer. Opt. Laser Technol..

[25] Piao JA, Li G, Piao ML, Kim N (2013). Full color holographic optical element fabrication for waveguide-type head mounted display using photopolymer. J. Opt. Soc. Korea.

[26] Shin CW (2021). Diffraction efficiency enhancement and optimization in full-color HOE using the inhibition characteristics of the photopolymer. Opt. Express.

[27] Chen Z, Yan S, Danesh C (2021). MicroLED technologies and applications: Characteristics, fabrication, progress, and challenges. J. Phys. D.

[28] Hsiang EL (2021). Prospects and challenges of mini-LED, OLED, and micro-LED displays. J. Soc. Inf. Disp..

[29] Blanche PA, Mahamat AH, Buoye E (2020). Thermal properties of bayfol® hx200 photopolymer. Materials.

[30] Sung CH, Chuang JZ (2010). The cell biology of vision. J. Cell Biol..

